# Comparison of Mean Platelet Counts in Preterm Infants with and without Retinopathy of Prematurity

**DOI:** 10.3390/ijerph18073783

**Published:** 2021-04-05

**Authors:** Zi Di Lim, Edwin Pheng, Evelyn Tai Li Min, Hans Van Rostenberghe, Ismail Shatriah

**Affiliations:** 1 Department of Ophthalmology and Visual Science, School of Medical Sciences, Universiti Sains Malaysia, Kubang Kerian 16150, Kelantan, Malaysia; limzidi@hotmail.com (Z.D.L.); edwinpcm@hotmail.com (E.P.); 2 Hospital Universiti Sains Malaysia, Jalan Raja Perempuan Zainab 2, Kota Bharu Kelantan 16150, Malaysia; hansvro@usm.my; 3 Department of Paediatrics, School of Medical Sciences, Universiti Sains Malaysia, Kubang Kerian 16150, Kelantan, Malaysia

**Keywords:** retinopathy of prematurity, platelet count, infant, premature

## Abstract

Platelets are a primary source of pro- and anti-angiogenic cytokines. However, the evidence of their role in retinopathy of prematurity (ROP) is controversial. This retrospective study aimed to compare mean weekly platelet counts between infants with and without ROP over the first 6 weeks of life. A total of 93 infants matched by gestational age and birth weight were recruited (31 with ROP, 62 without ROP). Weekly mean platelet counts and other related risk factors were documented. The repeated measure analysis of variance (ANOVA) and the repeated measure analysis of covariance (ANCOVA) were used to compare mean platelet counts over time between the two groups, with and without adjusting for confounders. We found significant differences in the weekly mean platelet counts of infants with and without ROP over the first 6 weeks of life (*p* = 0.002). These differences disappeared after adjusting for covariates (*p* = 0.489). Lower mean platelet counts in ROP infants are not directly related to ROP, but rather to the presence of other risk factors for ROP, such as culture-proven sepsis, blood transfusion and bronchopulmonary dysplasia.

## 1. Introduction

Retinopathy of prematurity (ROP) is a proliferative disorder of the retinal vasculature in premature infants. It is the leading cause of preventable blindness in children globally, with reported rates of blindness ranging from 10 to 40% [1,2,3]. The pathogenesis of ROP is multifactorial, with low birth weight, low gestational age and supplemental oxygenation being some of the implicated risk factors [4,5,6,7,8]. An imbalance of pro-angiogenic and anti-angiogenic cytokines is hypothesized to be the cause of retinal neovascularization and its sequelae in ROP. Platelets, being a source of these cytokines [9,10,11], may have a role in ROP development.

To date, the relationship between platelet counts and ROP remains poorly defined. Most studies observe an association between thrombocytopenia and ROP development or severity [12,13,14], while others find no association [15,16]; one recent study even documented that thrombocytosis is associated with ROP [17]. These differences may be attributed to differences in study methodology and statistical analysis, as the majority of these studies evaluated platelet levels as a qualitative variable. Quantification of platelet levels during the first 6 weeks of life may serve as an early indicator of ROP. Thus, this study aimed to compare mean weekly platelet counts between infants with and without ROP.

## 2. Materials and Methods

### 2.1. Study Population

This was a retrospective study among preterm infants admitted to Hospital Universiti Sains Malaysia from September 2016 to December 2019. A total of 93 infants were included in this study. The study was approved by the Human Research Ethics Committee of Universiti Sains Malaysia (USM/JEPeM/18090441). The conduct of the study followed the tenets of the Declaration of Helsinki. Inclusion criteria included all premature infants with a gestational age of less than 32 weeks and a birth weight less than 1.5 kg. Infants with significant congenital abnormalities, infants with ocular defects and infants who died before their ROP status was known were excluded. Each ROP case was individually matched to two non-ROP cases (1:2), with birth weight within 100 g and gestational age within 1 week of the study subjects. Subjects with extremely small gestational age or birth weight who could not be matched were excluded. Subjects with individual or parental history of platelet-related diseases (e.g., idiopathic thrombocytopenic purpura, hemangioma and trisomy diseases) were also excluded.

### 2.2. Data Collection

Data collected included birth weight, gestational age, gender, ROP stage, treatment and weekly mean platelet cell count from birth to week 6 of life. ROP stages were categorized into five stages as follows: Stage 1—demarcation line, i.e., a thin line separating the avascular retina anteriorly from the vascularized retina posteriorly; Stage 2—ridge, a raised demarcation line with width and height; Stage 3—extraretinal fibrovascular proliferation, in which neovascularization extends from the ridge to the vitreous; Stage 4—partial retinal detachment, extrafoveal (Stage 4a) or foveal (Stage 4b); Stage 5—total retinal detachment, in which total retinal detachment may be exudative or tractional [18]. Associated risk factors for ROP, including necrotizing enterocolitis, intraventricular hemorrhage, culture-proven sepsis, bronchopulmonary dysplasia, duration of supplemental oxygenation and total volume of blood transfusion were also documented.

### 2.3. Sample Size Calculation

Sample size calculation was determined using G Power Software 2010. The calculation was based on the repeated measure ANCOVA, with an effect size of 0.25, alpha of 0.05 and power of 0.8. This provided a sample size of 74. By allowing for 20% missing data, the total sample size was 93. A 1:2 ratio was used as ROP is much less common than the absence of ROP in a normal population.

### 2.4. Statistical Analysis

Statistical analyses were performed using IBM SPSS Statistics for Windows, Version 26.0 (IBM Corp, Armonk, NY, USA). A chi-square test was performed to determine the differences in categorical risk factors between infants with and without ROP. An independent *t*-test was used to determine the mean differences in numerical variables between the two groups. Forward stepwise multiple logistic regression was used to screen for confounding factors (i.e., other factors associated with ROP which may have influenced platelet counts). Repeated measure analysis of variance (ANOVA) was performed to determine the mean differences in weekly platelet counts from birth to week 6 of life between infants with and without ROP. The mean platelet count was then adjusted for confounding factors identified during logistic regression using the repeated measure analysis of covariance (ANCOVA). The repeated measure ANCOVA between groups was used to compare intergroup differences in mean platelet counts at specific times, and repeated measure ANCOVA within–between groups was used to compare changes in mean platelet count across time between groups. A comparison of mean platelet counts between different stages of ROP was similarly performed.

## 3. Results

A total of 93 infants were included in this study. The ROP group consisted of 31 infants and the group without ROP consisted of 62 infants. Of those with ROP, 8 (25.8%) had stage 1 ROP, 6 (19.3%) had stage 2 ROP, 16 (51.6%) had stage 3 ROP and only 1 (3.2%) had stage 4 ROP. The majority of these (19 infants, 61.3%) required treatment. Other clinical characteristics of our study subjects are shown in Table 1.

In univariate analysis, mean birth weight (OR 0.95, 95% CI 0.91–0.99), mean gestational age (OR 0.997, 95% CI 0.994–0.999), amount of blood transfusion (OR 1.04, 95% CI 1.02–1.05), duration of supplemental oxygenation (OR 1.16, 95% CI 1.05–1.29), culture-proven sepsis (OR 15.46, 95% CI 4.50–53.12) and bronchopulmonary dysplasia (OR 12.42, 95% CI 3.16–48.72) were statistically significant risk factors for the development of ROP. However, multivariable analysis identified only blood transfusion (OR, 1.03, 95%, CI 1.010–1.056), bronchopulmonary dysplasia (OR 6.41, 95% CI 1.28–31.93) and culture-proven sepsis (OR 8.79, 95% CI 2.09–36.93) as independent risk factors for ROP. Table 2 summarizes these results.

**Figure 1:**
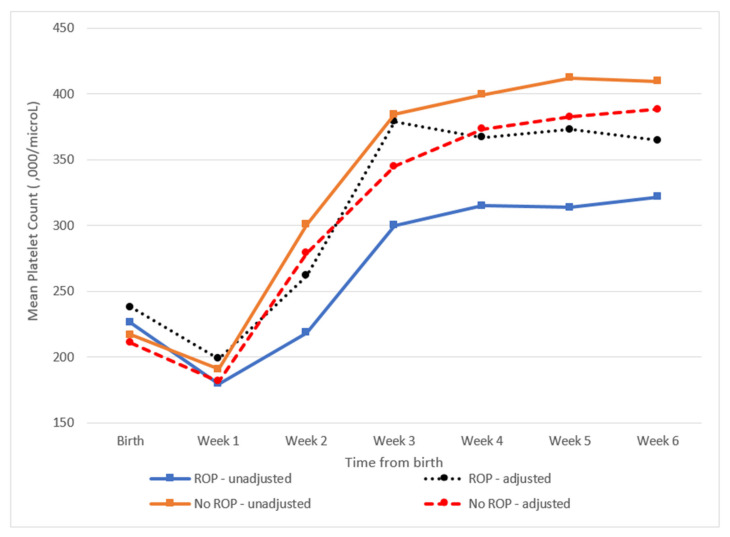
Mean platelet count in groups with and without retinopathy of prematurity (ROP) from birth to week 6 of life pre-and postadjustment for confounders (culture-proven sepsis, blood transfusion and bronchopulmonary dysplasia). This graphical depiction of the weekly mean platelet count in infants with and without ROP demonstrates that the difference between the two groups disappears after adjusting for covariates.

We found significant differences in the weekly mean platelet count between infants with and without ROP from week 2 to week 6 of life. However, repeated analysis with adjustments for covariates found no significant differences (Table 3). Overall, although there were significant intergroup differences in the change of mean platelet counts over the first 6 weeks (*p* = 0.002), this significance was lost after adjusting for confounders (*p* = 0.489). The unadjusted and adjusted mean platelet count in each group is illustrated in Figure 1. Similarly, a comparison of weekly mean platelet counts between the different stages of ROP revealed significant differences, although this significance once again disappeared after adjusting for covariates (*p* = 0.926 ) (Table 4).

## 4. Discussion

Advances in neonatal care have contributed to improved survival of preterm infants, particularly in technologically developed regions [18,19]. The incidence of ROP in high-income nations is decreasing, while in poorer regions, the converse is true [2,20]. ROP demographics and risk factors appear to vary worldwide, with severe ROP occurring even in more mature infants in low- and middle-income countries [1,21]. Identifying risk factors for ROP may enhance the efficacy of screening programs. Various studies have attempted to clarify the relationship between platelet counts and ROP [12,13,14,15,16,19,20,21,22,23] (Table 5). To our best knowledge, this is the first study to document that adjusted weekly mean platelet counts over the first 6 weeks of life have no direct relationship with ROP.

We observed that infants with ROP had significantly lower weekly unadjusted mean platelet counts from week 2 to week 6 of life, compared to their counterparts without ROP. Although this finding is consistent with the literature, limitations of previous approaches are that treating thrombocytopenia as a categorical variable (i.e., a single episode of platelet count less than 150,000/μL) may be too general of an approach and that failure to adjust for the effect of other parameters on platelets may confound the results [12,13,14]. Our study found that after adjusting for confounders, the intergroup significance between the differences in mean platelet counts disappeared. This is in keeping with the results of Korkmaz et al., who found no difference in mean platelet counts between ROP and control groups [16].

The change in mean platelet counts after adjustment may be attributed to the effect of the confounding factors (volume of blood transfusion, sepsis and presence of bronchopulmonary dysplasia) on ROP [24,25,26,27]. Sepsis is commonly known to be related to thrombocytopenia. Sepsis not only causes endothelial damage with resultant increased platelet activation, initiating a vicious cycle of subsequent platelet-mediated cytotoxic endothelium damage [28], but also causes bone marrow suppression, with consequent decreased platelet production [29]. A meta-analysis investigating the association between sepsis and ROP found that sepsis increases the risk of ROP [26]. This is consistent with our study. The hypothesized mechanisms by which sepsis results in ROP development are manifold. Firstly, sepsis could increase oxidative stress responses, leading to vascular cell degeneration and necrosis [30]. Besides that, microorganisms and toxins could cause damage to vascular endothelial cells, resulting in retinal nonperfusion [31]. We hypothesize that reduction in platelet counts indirectly reflects the ongoing septic process which predisposes these infants to ROP.

Preterm infants often have immature hematopoietic systems with inefficient erythropoiesis. The frequent phlebotomies required in these infants place them at greater risk of requiring blood transfusions [32]. Red blood cell transfusion has been shown to be an independent risk factor for the development of ROP, and this risk is proportionate to the volume transfused [25]. The increase in mean platelet counts after adjusting for blood transfusion suggests that infants with higher amounts of blood transfusions have lower platelet counts. Blood transfusions may cause platelet activation and aggregation [33,34], causing micro-occlusion of retinal vessels and resulting in platelet consumption from the bloodstream. Besides that, the shift in the ratio between adult and fetal hemoglobin, which has a higher affinity to oxygen, may cause relative hyperoxia [35], setting the stage for ROP development [36]. ROP severity has not been shown to differ between liberal and restrictive transfusion policies, so hemoglobin thresholds for transfusion should be based upon evidence-based recommendations [37,38].

In bronchopulmonary dysplasia, anomalous lung development with altered alveolar microvasculature results in inefficient gas exchange [39,40]. In fact, ventilation–perfusion abnormalities and prominent perfusion defects have been demonstrated in children up to a decade after development of this disease [41]. Animal studies show that chronic hypoxia decreases platelet counts [42,43]. Platelet deficiency may subsequently compound the disease severity, as platelet-derived growth factors are key components of normal alveolarization [44].

Platelet counts decrease within the first week of life and then increase over the next few weeks before they plateau. The increase in megakaryopoiesis is stimulated by increased thrombopoietin production during the first week of life [45]. Platelets being a primary source of vascular endothelial growth factor (VEGF) [11], we postulated that reduced platelet counts during the early phase of ROP development cause a decrease in systemic VEGF, reducing the amount of available VEGF in the eye and inhibiting normal angiogenesis. An alternate premise was that decreased platelet-derived anti-angiogenic stimuli may predispose an individual retina to neovascularization. The results of this study demonstrate that neither of these two hypotheses is likely; rather, the observed reduction in platelet levels in ROP occurs as an indirect effect of the other processes, which compounds the underlying susceptibility of these infants to ROP.

Our study’s findings may be extrapolated to similar populations, as we strictly adhered to guidelines for neonatal care, including policies for oxygen saturation monitoring, blood transfusion and ROP screening, and the mortality rate of the population of this study is comparable to that of other middle-income countries [46,47]. The strengths of this study are its use of weekly platelet counts in early life and utilization of statistical techniques which allowed adjustment for multiple confounders. There are a number of limitations in our study. First, this was a retrospective study. Second, data were obtained from a single center. Larger, prospective, multicenter studies with inclusion of angiogenic biomarkers may improve our understanding of the pathophysiological basis of this challenging disease.

## 5. Conclusions

Mean platelet counts over the first 6 weeks of life do not significantly differ between infants with and without ROP after adjusting for confounders. The lower mean platelet counts observed in ROP infants are attributed to the effect of ROP-associated factors, particularly culture-proven sepsis, blood transfusion and bronchopulmonary dysplasia. Clinicians should maintain a higher index of suspicion for ROP when screening premature infants with these risk factors.

## Figures and Tables

**Table 1:** Clinical characteristics of infants with and without retinopathy of prematurity (ROP).

Clinical Features	ROP n = 31	No ROP n = 62	*p* Value
Mean birth weight (g)	962.2 ± 167.9	1056.9 ± 173.8	0.014 *^,^^a^
Mean gestational age (weeks)	27.6 ± 1.9	28.5 ± 1.4	0.013 *^,^^a^
Respiratory distress syndrome	29 (93.5%)	59 (95.1%)	0.745 ^b^
Surfactant usage	27 (87.1%)	57 (91.9%)	0.457 ^b^
Intraventricular hemorrhage	17 (54.8%)	29 (46.7%)	0.463 ^b^
Culture-proven sepsis	16 (51.6%)	4 (6.4%)	<0.001 *^,^^b^
Pneumonia	10 (32.2%)	13 (20.9%)	0.234 ^b^
Necrotizing enterocolitis	5 (16.1%)	13 (20.9%)	0.578 ^b^
Congenital heart disease	10 (32.2%)	11 (17.7%)	0.114 ^b^
Blood transfusion (mL)	104.3 ± 62.3	48.7 ± 28.3	<0.001 *^,^^a^
Bronchopulmonary dysplasia	12 (38.7%)	4 (6.4%)	<0.001 *^,^^b^
Supplemental oxygenation (days)	14.4 ± 17.1	2.7 ± 3.7	<0.001 *^,^^a^
Multiple gestation	11 (35.4%)	13 (20.9%)	0.132 ^b^
Gender (male)	16 (51.6%)	40 (64.5%)	0.587 ^b^

^a^ Independent *t*-test was applied. ^b^ Pearson chi-square test was applied. * *p* < 0.05 indicates statistical significance.

**Table 2:** Factors associated with ROP.

Variables	Simple Logistic Regression			Multiple Logistic Regression		
	β	*p* Value	OR (95% CI)	β	*p* Value	OR (95% CI)
Mean birth weight (g)	−0.051	0.017 *	0.950 (0.911–0.991)			
Mean gestational age (days)	−0.003	0.017 *	0.997 (0.994–0.999)			
Blood transfusion (mL)	0.037	<0.001 *	1.038 (1.020–1.056)	0.031	0.003 *	1.030 (1.010–1.056)
Supplemental oxygenation (days)	0.154	0.003 *	1.167 (1.054–1.292)			
Intraventricular haemorrhage	0.323	0.464	1.382 (0.581–3.284)			
Culture-proven sepsis	2.739	<0.001 *	15.467 (4.503–53.128)	2.174	0.003 *	8.792 (2.093–36.936)
Pneumonia	0.585	0.585	1.795 (0.680–4.735)			
Necrotizing enterocolitis	−0.322	0.579	0.725 (0.233–2.257)			
Congenital heart disease	0.792	0.119	2.208 (0.816–5.976)			
Bronchopulmonary dysplasia	2.519	<0.001 *	12.421 (3.167–48.721)	1.859	0.023 *	6.416 (1.289–31.933)

* *p* < 0.05 indicates statistical significance.

**Table 3:** Comparison of mean platelet counts between infants with and without ROP based on time.

Mean Platelet Counts (000/ μL)	Adjusted Mean (95% CI)		*p* Value ^a^	*p* Value ^b^
	ROP	No ROP		
Without Covariates				
Birth	226.25 (205.34,247.17)	216.85 (202.06, 231.64)	0.468	0.002 *
Week 1	179.75 (160.77,198.74)	190.85 (177.42, 204.27)	0.346	
Week 2	218.34 (178.44,258.24)	300.72 (272.50, 328.93)	0.001 *	
Week 3	300.04 (249.08,351.01)	384.48 (348.44, 420.52)	0.009 *	
Week 4	314.93 (261.78, 368.09)	399.23 (361.64, 436.81)	0.012 *	
Week 5	313.82 (259.34, 368.30)	412.08 (373.56, 450.60)	0.004 *	
Week 6	321.71 (271.60, 371.82)	409.65 (374.22, 445.09)	0.005 *	
With covariates ^a^			*p* Value ^c^	*p* Value ^d^
Birth	238.00 (213.45, 262.54)	210.98 (195.04, 226.92)	0.107	0.489
Week 1	198.66 (176.93, 220.40)	181.39 (167.28, 195.51)	0.258	
Week 2	261.75 (216.48, 307.03)	279.01 (249.61, 308.41)	0.484	
Week 3	378.99 (325.57, 432.41)	345.01 (310.32, 379.70)	0.400	
Week 4	366.71 (307.06, 426.36)	373.34 (334.60, 412.08)	0.838	
Week 5	372.88 (310.78, 434.98)	382.55 (342.23, 422.88)	0.777	
Week 6	364.71 (310.50, 418.92)	388.15 (352.95, 423.35)	0.562	

^a^ Repeated measure ANOVA between groups. ^b^ Repeated measure ANOVA within–between groups (overall). ^c^ Repeated measure ANCOVA between groups, adjusted for culture-proven sepsis, blood transfusion and bronchopulmonary dysplasia. ^d^ Repeated measure ANCOVA within–between groups (overall), adjusted for culture-proven sepsis, blood transfusion and bronchopulmonary dysplasia.* *p* < 0.05 indicates statistical significance.

**Table 4:** Comparison of mean platelet counts between different stages of ROP.

Time	Mean Platelet Counts ± SD (000/μL)						
	No ROP N = 62	ROP Stage 1 N = 8	ROP Stage 2 N = 6	ROP Stage 3 or More N = 17	*p* Value ^a^	*p* Value ^b^	*p* Value ^c^
Birth	216.8 ± 57.5	219.1 ± 71.1	282.5 ± 36.8	209.7 ± 52.8	0.074	0.071	0.926
Week 1	190.8 ± 48.4	190.4 ± 61.0	213.9 ± 70.0	162.6 ± 56.3	0.180	0.561	
Week 2	300.7 ± 115.1	245.0 ± 134.7	274.3 ± 66.4	186.0 ± 92.9	0.004 *	0.852	
Week 3	384.4 ± 137.1	364.8 ± 181.4	370.2 ± 136.3	244.7 ± 130.3	0.006 *	0.846	
Week 4	399.2 ± 135.0	379.0 ± 194.5	334.9 ± 111.4	277.7 ± 180.4	0.041 *	0.887	
Week 5	412.0 ± 155.7	438.4 ± 178.3	317.3 ± 97.3	253.9 ± 107.9	0.002 *	0.258	
Week 6	409.6 ± 142.0	367.1 ± 85.0	382.7 ± 125.1	278.7 ± 151.1	0.023 *	0.923	

^a^ Repeated measure ANOVA between groups. ^b^ Repeated measure ANCOVA between groups, adjusted for culture-proven sepsis, blood transfusion and bronchopulmonary dysplasia. ^c^ Repeated measure ANCOVA within–between groups (overall), adjusted for culture-proven sepsis, blood transfusion and bronchopulmonary dysplasia. * *p* < 0.05 indicates statistical significance.

**Table 5:** Summary of literature regarding platelet counts in ROP.

Name	Country	Year	Sample Size	Study	Result	Conclusions
Bourla et al. [15]	USA	2008	178	Retrospective	*p* = 0.689	No association between thrombocytopenia and ROP
Rastogi et al. [22]	USA	2011	286	Retrospective	*p* < 0.001	A >30% drop in platelet counts is associated with ROP
Jensen et al. [19]	USA	2011	161	Retrospective	OR 6.69, 95% CI 2.82–15.9	Thrombocytopenia is associated with severe ROP, primarily in zone 1
Lundgren et al. [20]	USA	2017	18	Retrospective	*p* < 0.001	Thrombocytopenia at the time of ROP diagnosis is associated with APROP development
Korkmaz et al. [16]	Turkey	2017	146	Retrospective	*p* > 0.05	Platelet counts do not differ between groups with and without ROP
Cakir et al. [14]	USA	2018	202	Retrospective	OR 2.97 95% CI (1.37–6.46)	Thrombocytopenia is independently associated with severe ROP
Sancak et al. [13]	Turkey	2018	182	Retrospective	OR 59.0, 95% CI 51.14–71.0	There is a significant association between thrombocytopenia and Type I ROP
Jensen et al. [12]	USA	2018	100	Retrospective	OR 2.8, 95% CI 1.4–5.6	Thrombocytopenia from birth to 34 weeks GA is associated with subsequent severe ROP
Lundgren et al. [21]	Sweden	2020	78	Prospective	*p* < 0.01	ROP requiring treatment had lower platelet counts than ROP not requiring treatment

ROP, retinopathy of prematurity; APROP, aggressive posterior retinopathy of prematurity; GA, Gestational age.

## Data Availability

The data presented in this study are available on request from the corresponding author.
